# Newborn resuscitation in Gombe State, northeastern Nigeria

**DOI:** 10.7189/jogh.08.020420

**Published:** 2018-12

**Authors:** Josephine LR Exley, Nasir Umar, Sarah Moxon, Adamu Umar Usman, Tanya Marchant

**Affiliations:** 1 Centre for Evaluation and Department of Social and Environmental Health Research, London School of Hygiene & Tropical Medical, London, UK; 2 Maternal, Adolescent, Reproductive and Child Health (MARCH) Centre and Department for Disease Control, London School of Hygiene & Tropical Medical, London, UK; 3 Maternal, Adolescent, Reproductive and Child Health (MARCH) Centre and Department of Infectious Disease Epidemiology, London School of Hygiene & Tropical Medical, London, UK; 4 Data Research and Mapping Consult, Abuja, Nigeria

## Abstract

**Background:**

Basic newborn resuscitation for babies not breathing at birth is a highly effective intervention and its scale-up identified as a top research priority. However, tracking progress on the scale-up and coverage of this intervention is compromised by limitations in measuring both the number of newborns receiving the intervention and the number of newborns requiring the intervention. Using data from a facility and birth attendant survey in Gombe State, Nigeria, we aimed to advance the measurement agenda by developing a proxy indicator defined as the “percent of newborns born in a facility with the potential to provide newborn resuscitation”.

**Methods:**

The indicator’s denominator was defined as: the total number of births in facilities during a defined time period (facility records). The numerator was constructed from the number of those births that occurred in appropriately equipped facilities (facility inventory), where a birth attendant demonstrated basic resuscitation competence (assessed by a simulation exercise). The proportion of facility-births that took place in a setting with the potential to provide newborn resuscitation was then calculated.

**Results:**

The analysis included 17 383 births that occurred during May-October 2015 in 117 primary and referral facilities surveyed in November 2015. Overall 81% of the facilities did not have all items of essential equipment required for resuscitation; the items of equipment least frequently present included a timing device and resuscitation bag with two sizes of neonatal face mask. Only 3% of 117 birth attendants interviewed demonstrated competence to undertake resuscitation, all of whom were classified as skilled attendants and worked in referral facilities. We found that 20% of the 17 383 births took place in a facility with the potential to provide lifesaving resuscitation care.

**Conclusions:**

The indicator definition of neonatal resuscitation presented here responds to the need to advance the measurement agenda for newborn care and importantly adjusts for the volume of births occurring in different facilities. Its application in this setting revealed substantial missed opportunities to providing lifesaving care and highlights the need for a greater focus on input as well as process quality in all levels of health facilities.

To achieve greater progress in reducing under five mortality, more focus is needed on improving survival in the early neonatal period, especially on the day of birth. Intrapartum insults (leading to babies who are born gasping or not breathing at birth) is a leading cause of death at birth [1,2]. Treatment includes basic neonatal resuscitation: stimulation of a newborn who does not start crying by drying and rubbing the baby’s back, followed by bag and mask ventilation if required [3]. This intervention has been demonstrated to be effective and low cost [4-7], and has been described as an essential basic newborn signal function for all health facilities [8]. Mortality models estimate that 212 439 neonatal deaths could be averted by 2030 if resuscitation was universally available in the 75 countries that accounted for 99 per cent of newborn deaths in 2014 [9]. This figure is likely to be a substantial underestimate given the high risk of babies born at term not breathing being misclassified as a stillbirth [1,7].

The scale-up of basic neonatal resuscitation has been identified as a key high impact intervention by the Every Newborn Action Plan [10]. However, the ability to track progress on the scale-up of the intervention is compromised as coverage data are not currently available and the intervention lacks a standard measurable indicator [9,11,12]. A coverage indicator is defined as the proportion of individuals who need an intervention who actually receive it. For the indicator proposed by the Every Newborn Action Plan “percent of newborns not breathing or gasping at birth who are resuscitated using a bag and mask” both the denominator and numerator have proved almost impossible to count. Measuring resuscitation coverage requires accurate capture of the number of newborns that require resuscitation actions at birth (the denominator) [9]. According to WHO, resuscitation should be initiated for all non-macerated newborns who are not breathing spontaneously following immediate drying at birth [3]. Instances where resuscitation does not occur may be underreported or misclassified, especially where providers are inappropriately equipped or trained to take appropriate actions, or are unable to identify babies that need resuscitation. Even where resuscitation does take place, poor reporting and recall bias may affect accurate reporting of this number (the numerator). As a consequence, this priority indicator is not possible to track in its current form.

In this manuscript we suggest that the measurement agenda for newborn resuscitation with a bag and mask can be advanced by defining an alternative actionable proxy indicator for facility based births. It is not anticipated that this intervention would be available for home births. This revised measurement approach could meet the needs of both continuous improvement efforts and for tracking progress.

We argue that all babies born in a health facility have the right to expect that resuscitation is possible should the need arise, and therefore present an actionable alternative defined as the “percent of newborns delivered in a facility with the potential to provide newborn resuscitation”. To illustrate this, we analysed facility-level data from Nigeria on both the readiness of facilities and the competence of birth attendants in facilities to provide basic resuscitation care. We link this data to the volume of facility births in the same facilities to estimate the proportion of facility births that took place in a facility with the potential to provide newborn resuscitation care. Disaggregation of the resulting measure identifies the missed opportunities to providing lifesaving care, and therefore provides actionable evidence for improving health outcomes.

## METHODS

### Study setting

A facility survey was carried out in November 2015 in Gombe state, Nigeria. Gombe state is located in northeastern Nigeria, and has poor maternal and neonatal health outcomes. The majority (68%) of women give birth at home and the neonatal mortality rate is high, estimated in 2016-17 to be 35 per 1000 live births [13]. The majority of the population live in rural areas [14] and care is delivered via a network of primary health care facilities, run by local government and the state primary health care development agency, and secondary health facilities (eg, general and specialist hospitals) run by the state government. The federal government is responsible for tertiary health care services (eg, teaching hospitals and federal medical centres) [15].

### Data collection

The context for this study is described elsewhere [16]. A state-wide random sample of 107 from approximately 500 government-owned primary health facilities across all 11 local government areas was drawn, and a census of all 18 referral health facilities in the State undertaken. The facility survey protocol was similar to a Service Availability and Readiness Assessment (SARA) [17] and included inputs data on infrastructure, equipment and supplies, availability of staff, supervision and management processes. Additionally, it recorded information on the volume of births in facilities through data extraction from facility registers on the number and outcomes of all births during the previous six months. Evidence of health worker processes was captured through a simulation activity as part of a larger Birth Attendant interview at each surveyed facility with the birth attendant who had conducted the most recent delivery recorded in the maternity register (or second to last if most recent was unavailable, and so on). In this setting birth attendants in facilities can include trained traditional birth attendants (TBA), community health extension workers (CHEW), midwives, nurses and medical doctors (although the latter are almost entirely absent at the primary level). Through this sampling method we aimed to represent an up-to-date snapshot of birth attendants as experienced by the women of the State, irrespective of the cadre of birth attendants employed. For the simulation, birth attendants were asked to simulate management of an asphyxiated newborn using a NeoNatalie doll [18]. The interviewer briefly demonstrated how the doll works and the birth attendants were allowed time to familiarise themselves with the doll and equipment provided (cloth/blanket, cord ties, suction apparatus, bag and masks, stethoscope and time piece). The interviewer then provided the following instructions: “Fatima has given birth to a 2800 g baby boy after a prolonged second stage of labour. This is her second pregnancy. Her first baby is still alive. But at this birth, Fatima’s newborn is blue and limp and does not breathe. You are the birth attendant. What are your next actions?”. This scenario was then followed-up with a statement “The baby is not responding after suctioning and rubbing. What will you do next?” The interviewer recorded the actions against a detailed pre-defined checklist adapted from the Helping Babies Breathe training manuals [19].

### Analysis

Analysis of the indicator “percent of newborns born in a facility with the potential to provide newborn resuscitation” was restricted to facilities that had recorded at least one delivery in the six months preceding the survey. Potential to provide resuscitation care was constructed from two measures (**Table 1**). First, we tabulated the availability in facilities of items specified by the Helping Babies Breathe framework as required for safely preparing for birth [20]: gloves, cloths, head covering, scissors, cord ties, suction device, bag and mask size 0 and 1, stethoscope and timer. We excluded cloths and head coverings from the tabulation as in this setting mothers are expected to provide these items rather than facilities keep them in stock. And second, we tabulated birth attendants’ competence to provide resuscitation care from the NeoNatalie demonstration data. We defined three key stages: (1) stimulates breathing (birth attendant clears the airway and stimulates baby by rubbing back); (2) clean cord cutting (birth attendant ties or clamps cord immediately, then cuts cord with clean blade or clean scissors); and (3) birth attendant initiates resuscitation (places newborn on back on a clean surface and checks the seal between mask and newborn’s face by ventilating two times and observing for the rise of the chest). Each stage was conditional on the previous stage, so to be classified as “competent” the birth attendant had to carry out all three stages. Both facility readiness and birth attendants’ competence tabulations were combined to create a facility-level indicator of “potential to resuscitate”.

**Table 1:** Definition of measures

Variable	Description
Population in need (denominator)	All deliveries that occurred in the last six months in the included facilities.
Facility readiness	All pieces of equipment required to undertake resuscitation was available and functioning. This includes:
	-sterile scissors or blade
	-disposable gloves
	-a clamp or umbilical tie
	-suction device
	-mask size 0 and 1
	-stethoscope
	-timing device
Birth attendants’ competence	Birth attendant demonstrated three initial actions during the NeoNatalie demonstration:
	-Stage 1: stimulates breathing; *[baby still not responding]*
	-Stage 2: clean cord cutting
	-Stage 3: initiate resuscitation with bag and mask
Potential to provide resuscitation care (numerator)	Facility with both the equipment available and function AND a birth attendant who demonstrated competency to undertake resuscitation.

The facility data set was then reshaped using the expand command in STATA on the count of the number of births recorded in each facility register in the six months before the facility survey, so that each birth was now recorded as a single observation in the data set. From this reshaped data we calculated the proportion of births in the sample that took place in a facility: (1) with the equipment available; (2) where a birth attendant had demonstrated competence; and (3) with the potential to provide resuscitation care, by dividing the number of births who satisfied the condition by the total number of births in the sample. Tabulations were carried out for both primary and referral levels, and for birth attendants classified as either skilled (doctor, nurse, midwife) or unskilled (TBA, CHEW and “other”).

Missed opportunities in readiness and competence were identified from the absolute attrition in proportion between the sample population and each of the measures (**Table 1**). The analysis was performed in Stata version 14 (Stata Inc, College Station, TX, USA).

## RESULTS

Data were collected from a total of 125 facilities, of which 117 recorded at least one delivery in the preceding six months (May-October 2015) and were included in this analysis. The majority, 86% (101/117), were primary care facilities (**Table 2**). The number of births recorded in the maternity registers in the last six months ranged from two to 3018 births per facility. On average, referral facilities handled a higher volume of deliveries than primary care facilities; 488.8 births (standard deviation (SD) 739.7) compared to 94.7 (SD 97.6), respectively. Of the 117 birth attendants interviewed who completed the NeoNatalie demonstration, 16% (19/117) were internationally classified as skilled (nurses, midwives or doctors), with large differences by level of facility (**Table 2**).

**Table 2:** Number of facilities and birth attendants by facility level

	N (column %)		
**All**	**Primary**	**Referral**
Facilities surveyed with at least one birth event in the preceding 6 months	117 (100%)	101 (100%)	16 (100%)
Number of birth attendants internationally categorised as skilled (doctor, nurse, midwife)	19 (16%)	7 (7%)	12 (75%)

Marked differences in the availability of equipment was observed between the different items surveyed (**Table 3**). Stethoscope, disposable gloves, sterile cord cutter and clamp/umbilical tie were almost universally available, while a working timing device was available in approximately two-thirds of facilities, and both sizes of bag and mask for resuscitation were available in only one-third of facilities (**Table 3**). Overall 19% (22/117) of the included facilities had all items of equipment required to undertake resuscitation present and functioning. Half of referral facilities had all items of equipment compared to 14% (14/100) of primary facilities.

**Table 3:** Facility readiness and birth attendances’ competence to provide resuscitation care

	All (N = 117)	Primary (N = 101)	Referral (N = 16)
**Facility readiness (equipment available and functioning):**			
Stethoscope	116 (99%)	100 (99%)	16 (100%)
Working timing device	73 (62%)	61 (60%)	12 (75%)
Sterile cord cutter	113 (97%)	97 (96%)	16 (100%)
Clamp/umbilical tie	108 (92%)	92 (91%)	16 (100%)
Disposable gloves	115 (98%)	100 (99%)	15 (94%)
Suction device	105 (90%)	89 (88%)	16 (100%)
Bag and mask (size 0)	46 (39%)	36 (36%)	10 (63%)
Bag and mask (size 1)	54 (46%)	43 (43%)	11 (69%)
***All items available***	***22 (19%)***	***14 (14%)***	***8 (50%)***
**Birth attendants’ competence:**			
1. Stimulates breathing	29 (25%)	21 (21%)	8 (50%)
2. Clean cord cutting	41 (35%)	33 (33%)	8 (50%)
3. Initiates bag and mask	7 (6%)	4 (4%)	3 (19%)
***Completes all 3 stages***	***3 (3%)***	***0***	***3 (19%)***

The proportion of birth attendants undertaking each stage of resuscitation was low and only 6% (7/117) of all birth attendants undertook stage 3 ‘initiates resuscitation with bag and mask’ (**Table 3**). A consistently higher proportion of birth attendants in referral facilities completed the various stages of resuscitation. Overall, 3% (3/117) of birth attendants (all skilled attendants from referral facilities) completed all three stages and thus were considered to have demonstrated competence to undertake resuscitation.

In combination, only two facilities, both referral facilities, had all items of equipment present and a birth attendant who had demonstrated competency to undertake resuscitation.

A total of 17 383 deliveries were recorded in the last six months across the 117 facilities surveyed, just under half of which (45%) were in the 16 referral facilities. Both the availability of functioning equipment and demonstrated birth attendants’ competence were found to contribute to missed opportunities to providing resuscitation care at birth (**Table 4**). Overall, 43% of these births occurred in a facility with all required equipment and 20% where the birth attendant also demonstrated competence to undertake resuscitation. There was a stark difference by level of facility; none of the 9563 births in a primary care facility had the potential to receive resuscitation care (ie, all required equipment available and a birth attendant who had demonstrated competence), while 45% of the 7820 births in referral facilities had the potential to receive resuscitation care.

**Table 4:** Number of births in health facilities with the potential to resuscitate a newborn at birth

	All	Primary	Referral
**Total number of births in the preceding six months**	17 383 (100%)	9563 (100%)	7820 (100%)
**Number of births where the following resuscitation conditions were met:**			
Facility readiness	7543 (43%)	2012 (21%)	5522 (71%)
Facility readiness and stage 1 of resuscitation	3917 (23%)	245 (3%)	3672 (47%)
Facility readiness and stages 1 & 2 of resuscitation	3588 (21%)	19 (0.2%)	3569 (46%)
Potential to provide resuscitation care (facility readiness and stages 1, 2 & 3 of resuscitation)	3537 (20%)	0	3537 (45%)

## DISCUSSION

In the absence of a standard measurable indicator and accurate data on the number of newborns that require resuscitation at birth, advances in measurement that reflect health system quality and can inform action in middle and low income settings are needed [9,11,12,21]. Using data collected in Gombe state, Nigeria, this study advances the measurement agenda by defining an actionable proxy indicator for newborn resuscitation as “the proportion of facility births that occurred in a setting with the potential to provide resuscitation care”. This new measurement approach provides evidence for both benchmarking facility births and continuous improvement efforts.

This study revealed substantial missed opportunities to provide lifesaving care at birth in Gombe state; 80% of births in this sample occurred in a facility without the potential to provide resuscitation care if required. Assuming that up to 10% of all facility births may require some degree of resuscitation [4], this translates into missed opportunities for as many as 1391 newborns in this sample. Consistent with existing literature, the analysis highlighted that both the availability of functioning equipment required for resuscitation, particularly bag and correct size masks, was low and that birth attendants lacked the skills to provide effective newborn resuscitation [4,22,23].

The correct use of a bag and mask was observed only amongst skilled birth attendants in referral facilities; most skilled birth attendants interviewed were not able to demonstrate all three stages of resuscitation, suggesting that the presence of a skilled attendant alone may not be sufficient to ensure high quality resuscitation care. An evaluation of Helping Babies Breathe training for nurse midwives and physicians in India and Kenya found only 5% of participants could ventilate a newborn mannequin correctly before training [24]. Training led to significant improvements in completion rates but the study demonstrates that the retention of skills is challenging; six months after receiving training completion rates fell by 16%. Maintaining high levels of readiness for neonatal resuscitation is likely to be a key challenge for birth attendants in this study setting as the volume of births was on average low [25]. Half of facilities handled fewer than 80 births in the preceding six months. Taken together with our finding that less than half of facilities had access to a bag and mask suggests that all birth attendants in this study, regardless of cadre, did not have sufficient experience of performing resuscitation.

Our findings suggest that giving birth in a health facility might not prevent avoidable newborn deaths and highlight the need for a greater focus on input and process quality in health facilities [26]. This is not unique to newborn care; the quality of essential primary-care services for women and children has been found to be weak in similar settings [23]. Our findings should prompt an examination of supply management strategies and actions for improving access to birth attendants with the required skills and support to maintain skills. This could include pre-service but importantly in-service training, supervision and mentoring by more highly skilled staff, in addition to the strategic distribution of the workforce [22].

### Limitations

There are a number of limitations to the proposed indicator that are worth considering. Data are restricted to facility based births and as such do not provide an indication of missed opportunities at the population level. However, given that in this setting neonatal resuscitation care with a bag and mask is only provided in facilities we believe examining facility potential to provide lifesaving care is justified. The proposed denominator is based on the assumption that all births could potentially require resuscitation care rather than restricting to the number that actually need resuscitation actions at birth. Given the challenge of accurately capturing the number of newborns that require resuscitation [1,7], we believe measuring facility preparedness to provide lifesaving care could provide an actionable alternative for improving health outcomes, as all newborns born in a health facility have the right to expect that resuscitation is possible should the need arise.

Applying facility readiness and birth attendants’ competency based on the day of survey to all births in the last six months assumes that readiness is static and that the birth attendant interviewed was representative of all staff taking deliveries in the facilities. In reality a facility may be resuscitation ready one day but not the next. Simulation observations are susceptible to both observer and responder biases which cannot be discounted despite the provision of training for interviewers and the use of supervision protocols throughout.

Finally, the data presented were specifically collected as part of the IDEAS study. Data on the availability and functioning of basic equipment for neonatal resuscitation, including smaller-sized face masks and resuscitation bag in the labour and delivery room are captured in logistic management information systems including the Demographic and Health Survey Programme’s Service Provision Assessment (SPA), the WHO SARA and the emergency obstetric and neonatal care (EmONC) assessments [27]. Whilst these surveys also collect data on whether the intervention is regularly performed, data on birth attendant’s competence is unlikely to be available without improvements in supportive supervision and tools for monitoring quality of service delivery. The challenge moving forward is to ensure routine systems harmonise the indicators being collected to allow comparisons across settings and, longer term, to establish how information systems can integrate quality measures, support ongoing service improvement and enhance the potential for life saving care.

## CONCLUSIONS

The proxy indicator definition of neonatal resuscitation presented here - that speaks to the potential of every newborn to receive life-saving care – responds to the need to advance the measurement agenda. Its application in this setting identified substantial missed opportunities to provide lifesaving care and highlighted the need to improve the availability of essential equipment and ensure that birth attendants acquire and maintain their skills to provide high quality newborn care.
